# SARS-CoV-2 Epitope Mapping on Microarrays Highlights Strong Immune-Response to N Protein Region

**DOI:** 10.3390/vaccines9010035

**Published:** 2021-01-11

**Authors:** Angelo Musicò, Roberto Frigerio, Alessandro Mussida, Luisa Barzon, Alessandro Sinigaglia, Silvia Riccetti, Federico Gobbi, Chiara Piubelli, Greta Bergamaschi, Marcella Chiari, Alessandro Gori, Marina Cretich

**Affiliations:** 1 National Research Council of Italy, Istituto di Scienze e Tecnologie Chimiche “Giulio Natta” (SCITEC-CNR), Via Mario Bianco 9, 20131 Milano, Italy; angelo.musico94@gmail.com (A.M.); roberto.frigerio94@gmail.com (R.F.); alessandro.mussida@scitec.cnr.it (A.M.); greta.bergamaschi@cnr.it (G.B.); marcella.chiari@cnr.it (M.C.); 2 Department of Molecular Medicine, University of Padova, Via A. Gabelli 63, 35121 Padova, Italy; luisa.barzon@unipd.it (L.B.); alessandro.sinigaglia@unipd.it (A.S.); silvia.riccetti@unipd.it (S.R.); 3 Department of Infectious-Tropical Diseases and Microbiology, IRCCS Sacro Cuore Don Calabria Hospital, Via Don A. Sempreboni, 5-37024 Negrar di Valpolicella, Italy; federico.gobbi@sacrocuore.it (F.G.); chiara.piubelli@sacrocuore.it (C.P.)

**Keywords:** SARS-CoV-2, Covid-19, serological test, epitope mapping, peptide microarrays, click chemistry

## Abstract

A workflow for rapid SARS-CoV-2 epitope discovery on peptide microarrays is herein reported. The process started with a proteome-wide screening of immunoreactivity based on the use of a high-density microarray followed by a refinement and validation phase on a restricted panel of probes using microarrays with tailored peptide immobilization through a click-based strategy. Progressively larger, independent cohorts of Covid-19 positive sera were tested in the refinement processes, leading to the identification of immunodominant regions on SARS-CoV-2 spike (S), nucleocapsid (N) protein and Orf1ab polyprotein. A summary study testing 50 serum samples highlighted an epitope of the N protein (region 155–71) providing good diagnostic performance in discriminating Covid-19 positive vs. healthy individuals. Using this epitope, 92% sensitivity and 100% specificity were reached for IgG detection in Covid-19 samples, and no cross-reactivity with common cold coronaviruses was detected. Likewise, IgM immunoreactivity in samples collected within the first month after symptoms onset showed discrimination ability. Overall, epitope 155–171 from N protein represents a promising candidate for further development and rapid implementation in serological tests.

## 1. Introduction

In the frame of the ongoing pandemic caused by severe acute respiratory syndrome coronavirus 2 (SARS-CoV-2), reliable diagnostic and prognostic tools are constantly needed for the appropriate management of patients, to guide the development and implementation of surveillance and prevention measures, and to assess the efficacy of therapeutic interventions and vaccines. While molecular and antigenic testing of respiratory samples are used for the diagnosis of acute SARS-CoV-2 infection, serology testing allows the identification of individuals with recent or past exposure to SARS-CoV-2. Serological tests can be combined with molecular testing to improve the sensitivity and specificity of diagnosis [1] and represent key instruments for epidemiological surveillance to guide public health interventions [2]. Data from representative and reliable seroprevalence studies will greatly support decision-making practices in terms of physical distancing and other restrictions. However, the use of seroprevalence data to inform policymaking strictly depends on the accuracy and reliability of tests. Yet, currently available serological assays are biased by false-positive and false-negative results as pointed out by sub-optimal sensitivity and specificity performance (https://www.fda.gov/medical-devices/emergency-situations-medical-devices/eua-authorized-serology-test-performance).

To date, the majority of the serological studies confirmed a general humoral response upon SARS-CoV-2 infection against spike (S) and nucleocapsid (N) proteins, observed as early as the 4th day after symptom onset, when SARS-CoV-2-specific IgG and IgM antibodies appear simultaneously or sequentially and plateau within 6 days after seroconversion [3,4]. Most current immunoassays use recombinant coronavirus proteins: spike (S), which is exposed on the surface of the viral particle, and nucleocapsid (N), which is highly expressed during infection [3,5]. Full-length recombinant antigens, however, in addition to high costs and storage constraints, may suffer from stability problems, batch-to-batch variations and in some cases may give rise to cross-reactivity leading to ambiguous detection outcomes. This might be the case for SARS-CoV-2 when using proteins sharing high homology with genetically similar, co-circulating, human coronaviruses (hCoV) that are responsible for common cold [6,7]. Studies towards fine dissection of the specific regions on the viral proteins recognized by virus-specific antibodies have been recently published focusing on the S protein [6,8,9,10,11]. Moreover, detailed overviews of the epitope landscape within SARS-CoV-2 confirmed interest on the N protein epitopes as valuable markers [12,13]. In a seminal work by Shrock et al. [12], the authors used a breakthrough technology, i.e., VirScan, to identify several epitope candidates from the SARS-CoV-2 antigens. Alternatively, peptide microarrays represent appealing tools, as they allow the rapid screening of hundreds to thousands different peptides immobilized in spatially ordered patterns of spots on solid supports [14,15,16]. This approach was used to provide a first landscape of B-cell epitopes in the serum of 10 coronavirus disease of 2019 (Covid-19) patients [13].

**Figure 1:**
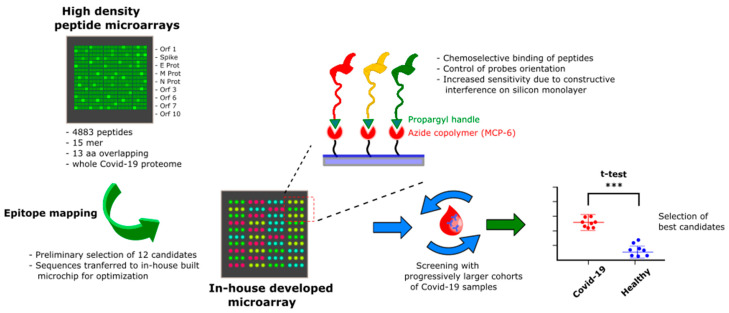
Overview of the probe selection workflow. A high-density peptide microarray displaying the whole SARS-CoV-2 proteome was probed for immunoreactivity with Covid-19 serum samples leading to the selection of 12 peptide hits. The most promising candidates were then transferred to a low-density microarray platform for site-selective and oriented peptide immobilization and validated with progressively larger and independent cohorts of patients to select the most sensitive and specific immunoreactive peptide probes at a statistically significant level (expressed by ***).

Herein we report on the further validation of SARS-CoV-2 linear epitopes, that were screened with serum samples collected from 50 Covid-19 patients at 1–5 months after symptom onset, including 5 patients with sera collected at different time-points after symptom onset. Our work was guided by a proteome-wide peptide microarray that was implemented into our previously developed silicon based microarray platform for site-selective and oriented peptide immobilization. [17,18]. In the process, we performed an iterative refinement of peptide hits by using progressively larger and independent cohorts of patients; such approach resulted in the restriction of the panel of effectively discriminating probes (Figure 1), with the best performing linear epitope belonging to the N protein (region 155–171). Interestingly, while based on a rapid screening platform, our results match the ones by Shrock et al. [12] and integrate the information obtained by others [13] in smaller and non-independent patients cohorts. This knowledge could improve our understanding of diverse disease outcomes and inform the development of improved diagnostics, vaccines, and antibody-based therapies [12,19].

## 2. Materials and Methods 

### 2.1. Serum Samples

Serum samples were collected from subjects with diagnosis of SARS-CoV-2 infection confirmed by real-time RT-PCR testing of nasopharyngeal swabs. The presence and titer of SARS-CoV-2-neutralizing antibodies in serum was determined by an in-house virus microneutralization assay, which is considered the reference test in coronavirus serology. Disease severity was classified as (1) mild, non-hospitalized, (2) moderate, hospitalized, and (3) severe, admitted to the intensive care unit. Study subjects included 92 patients with mild or moderate Covid-19 and 15 patients with severe Covid-19. Leftover serum samples collected from 50 healthy subjects before December 2018 were used as control.

Specimens were stored at −20 °C until use. The study was approved by the ethic committee of IRCCS Sacro Cuore Don Calabria Hospital (protocol n. 2998CESC) and study subjects provided written informed consent.

### 2.2. Proteome-Wide Microarray

A PEPperCHIP^®^ Peptide Microarrays slides with 1 subarray, was used where each peptide sequence is composed by 15 aminoacids with an overlap of 13 residues. Serum samples were incubated dynamically over night at 4 °C, diluted 1:50 in incubation buffer and subsequent protocol steps performed according to manufacturer’s instructions. Secondary antibody (Anti-Human IgG-Cy3, Jackson ImmunoReserarch, Cambridge, UK) was used for fluorescence detection and microarrays scanned by TECAN power scanner at 10% laser intensity and 50% gain.

### 2.3. Peptide Synthesis

All peptides were synthesized in house by stepwise microwave-assisted Fmoc-SPPS on a Biotage ALSTRA Initiator+ peptide synthesizer (Uppsala, Sweden) according to well-established protocols. Briefly, peptides were assembled on a 2-CTC resin (0.5 mmol/g loading) swelled in DCM/NMP 3:1. Chain elongation was performed by iterative cycles of amino acids coupling (using Oxyma/DIC (IRIS Biotech, Marktredwitz, DE) as activators) and Fmoc-deprotection using a 20% piperidine solution in DMF. Coupling steps were performed for 20 min at 50 °C. Fmoc-deprotection steps were performed by treatment with 20% piperidine solution at 40 °C for 10 min. Following each coupling and deprotection step, peptidyl-resin was washed with DMF. Upon complete chain assembly, peptides were cleaved from the resin using a 2.5% TIS, 2.5% thioanisole, 2.5% water, 92.5% TFA mixture. Crude peptides were then purified by preparative RP-HPLC (Shimadzu, Kyoto, Japan). Finally, purified peptides were lyophilized and stored at −20 °C.

### 2.4. Peptide Microarray

Silicon slides (SVM, Sunnyvale, CA, USA) were coated by MCP6 (Lucidant Polymers) as follows: slides were treated with oxygen plasma for 15 min. MCP6 was dissolved in DI water to final concentration of 2% *w/v* and then diluted with ammonium sulphate solution 40% at ratio 1:1. Subsequently, silicon slides were immersed into the polymer solution for 30 min, then washed with DI water, dried under nitrogen steam and cured for 15 min under vacuum at 80 °C.

Peptides were first dissolved in DMSO to 1 mM stock solution and then diluted to the final spotting concentration of 50 µM in printing buffer composed by 25 mM Na/Acetate pH 4.8, 15 mM trehalose, CuSO_4_ 100 µM, ascorbic acid 6.25 µM and THPTA 400 µM as previously reported in [20,21]. Slides were spotted using a non-contact Spotter S12 (Scienion Co., Berlin, Germany), in each subarray we used as positive control a peptide sequence derived from poliomyelitis virus. Printed slides were placed in a humid chamber overnight at room temperature. Then they were blocked in 2 mM EDTA water solution for 1 h, washed with water, and dried under a stream of nitrogen.

### 2.5. Bioassays

All samples were diluted 1:50 in incubation buffer with 1% BSA and incubated dynamically for 1 h at room temperature. Then, slides were washed 3 times for 1 min with washing buffer. The second incubation was performed with secondary antibody (Anti-Human IgG-Cy3, Jackson ImmunoReserarch; Anti-Human IgM-Cy5, Invitrogen, Waltham, MA, USA) diluted in ratio 1:1000 in incubation buffer with 1% BSA. Finally, slides were washed, dried and analyzed on a TECAN power scanner 10% laser intensity and 50% gain.

The full recombinant N antigen for immunoreactivity testing was a kind gift from LifeLineLab, (Pomezia, Italy). The protein was immobilized on copoly(DMA-NAS-MAPS) coated silicon slides and tested according to previously developed protocols [22,23].

## 3. Results and Discussion

### 3.1. SARS-CoV-2 Whole-Proteome Epitope Mapping

Preliminary data on IgG response in sera of Covid-19 patients were generated using high-density peptide array made of 4883 different peptides, 15 amino acids long, 13 amino acids overlap (PEPperCHIP^®^ SARS-CoV-2 Proteome Microarray) displaying sequences of ORF1ab polyprotein, surface glycoprotein (S), ORF3a protein, envelope protein (E), membrane glycoprotein (M), ORF6 protein, ORF7a protein, ORF8 protein, nucleocapsid phosphoprotein (N) and ORF10. Serum samples were collected from a group of seven Covid-19 patients, aged 68–96 years, at about 30 days after the onset of symptoms. In these patients, who had mild or moderate Covid-19, diagnosis of SARS-CoV-2 infection was confirmed by molecular testing of nasopharyngeal swabs. The presence of SARS-CoV-2-specific antibodies in corresponding serum samples was demonstrated by virus microneutralization assay. Table 1 reports demographic and clinical data of the Covid-19 patients tested in this study.

The whole-proteome peptide microarray screening was performed according to manufacturer instructions (see Section 2).

Epitope mapping results were analyzed in terms of fluorescence intensity of IgG immune-reactivity of Covid-19 positive sera and discrimination with the healthy control group by the unpaired *t* test (significative *p* < 0.05). Several immunoreactive regions were identified on nucleocapsid (N) protein, spike (S) protein and ORF1ab polyprotein. No reactivity was observed on the sequences from the other proteins displayed on the microarray.

**Figure 2:**
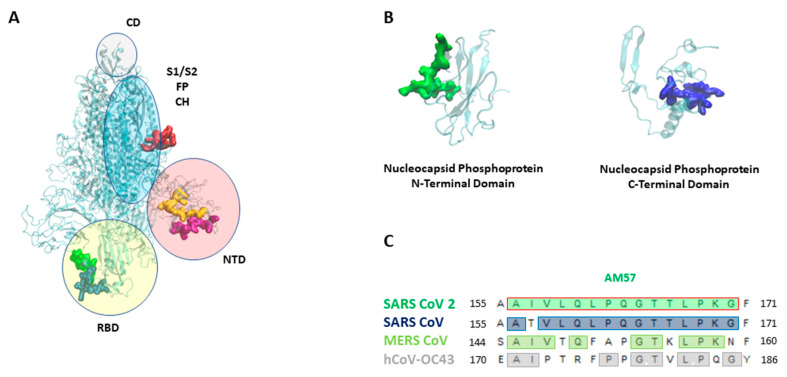
(**A**) SARS-CoV 2 Spike (S) protein with its domains [19] (NTD: n terminal domain, S1 e S2: furin cleavage site, CD: connecting domain, HR2: heptad repeat 2, FP: fusion peptide, CH: central helix). Epitopes are highlighted as follows: AM55 in red; AM50 in yellow; AM64 in purple; AM 63 in blue, AM 49 in green. (**B**) SARS-CoV 2 nucleocapsid (N) protein, the N-terminal and C-terminal domains. Epitopes are highlighted as follows: AM57 in green, AM66 in blue. (**C**) AM57 sequence alignment on SARS CoV 2, SARS CoV, MERS CoV, hCoV-OC43.

To define a set of immunodominant epitopes for further characterization, we selected the 10 most reactive antigenic regions corresponding to at least three contiguous overlapping peptides that showed a fluorescence intensity statistically different from the one detected in healthy controls. Two more immunoreactive sequences fulfilling the discrimination criteria were added to the previously selected, even if fluorescence was detectable only on a single sequence and not in the adjacent overlapping peptides. The selection of the 12 most reactive aminoacidic sequences is reported in Table 2; the resulting linear epitopes for protein S and N are mapped within antigen structures in Figure 2A,B.

Notably, our data on SARS-CoV-2 proteins primarily targeted by Covid-19 patient antibodies (proteins S, N and ORF1ab) are in accordance with a detailed exploration of the humoral response in Covid-19 sera recently performed by a programmable phage-display immunoprecipitation and sequencing technology [12].

### 3.2. Epitopes Validation Screening

**Figure 3:**
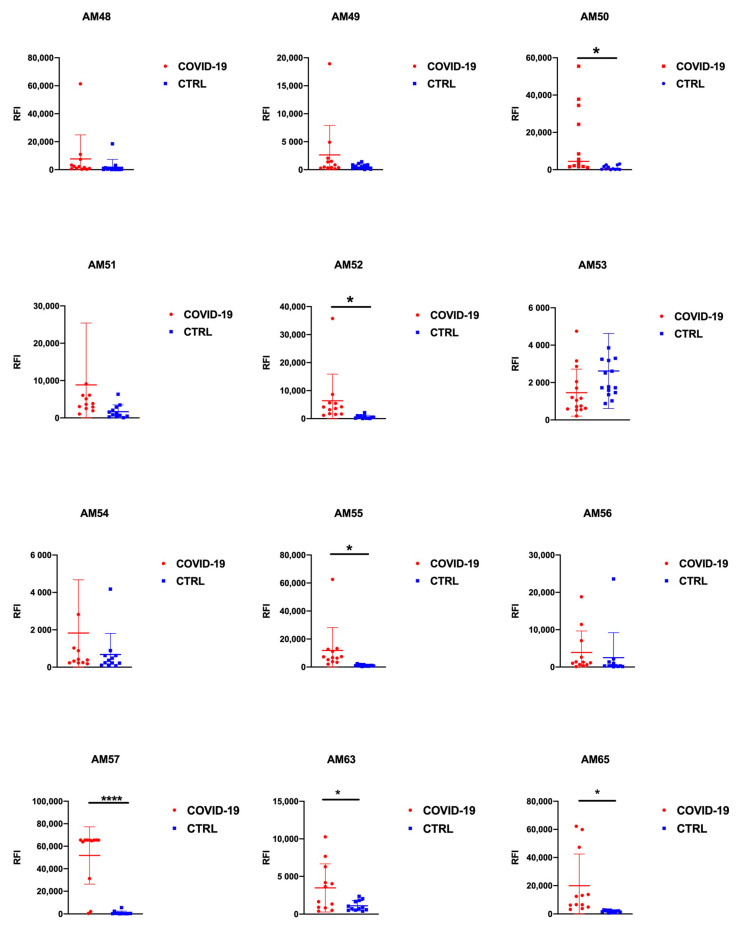
Unpaired *t*-test results for the peptide specific IgG. Peptide arrays displaying twelve peptide probes were probed with sera (N = 12) from COVID-19 patients and control patients (N = 12). Significative: *p* < 0.05; * *p* < 0.05; ** *p* < 0.01; *** *p* < 0.001; **** *p* < 0.0001.

More focused validation screenings on the 12 hit epitopes derived from the high-density microarray were then performed by progressively increasing the number of patients tested and using independent cohorts. In these experiments, peptides were in-house synthesized including an azide handle, in order to apply a ‘click’ chemoselective strategy for peptide binding to polymer-modified microarrays slides (Figure 1) [17]. This strategy was previously shown to allow for fine control of ligand orientation and probe surface density, which correlated with improved assay performances [17,20,21]. The initial set of 12 probes was first screened for IgG immunoreactivity against sera collected from a cohort of *N* = 12 Covid-19 patients at about 30 days after symptom onset vs. *N* = 12 pre- Covid-19 controls (Figure 3) and their discrimination performance evaluated by the unpaired *t* test (Table 2). Six out of the twelve putative epitopes identified by the initial screening showed ability to discriminate the two sample groups at a statistically relevant level: AM50, AM55 and AM63 from the S protein, AM52 and AM65 from Orf1ab polyprotein and AM57 form the N protein.

The 6 sequences providing a *p* value < 0.05 were re-evaluated in a second screening using a larger independent cohort of *N* = 28 patients, who were evaluated at 1–5 months after symptom onset (Figure S1). Again, testing a different and larger cohort of samples reduced the number of hit epitopes because a statistically significant ability to discriminate Covid-19 patients was found only in 3 out of 6 sequences: AM50 (S protein), AM65 (Orf1ab polyprotein) and AM57 located on the N protein whereas a loss of discrimination ability for probes AM52, AM55 and AM63 was observed (Table 2).

### 3.3. N Protein Epitope 155-171 (AM57) Shows Promising Diagnostic Potential

Epitope AM57 from the N protein consistently showed a high immunoreactivity, negligible response in healthy controls along all the screenings and the best diagnostic performance (*p* value < 0.0001). We also performed alignment of AM57 sequence against SARS-CoV, MERS-CoV and hCoV-OC43 (www.bioinformatics.org), (Figure 2C), to assess whether it is conserved among the same virus family. Importantly, in light of possible pre-existing cross-reactive responses in healthy controls, AM57 (Figure 2C) revealed only 53% amino acid identity with the corresponding sequence from the N protein of hCoV-OC43, responsible for the common cold. The high conservation with SARS-CoV suggests that cross-reactivity is likely, and the probe could actually serve for dual detection. However, since SARS-CoV has no longer propagated for many years, the antibodies titer of currently screened samples would not be likely detectable.

**Figure 4:**
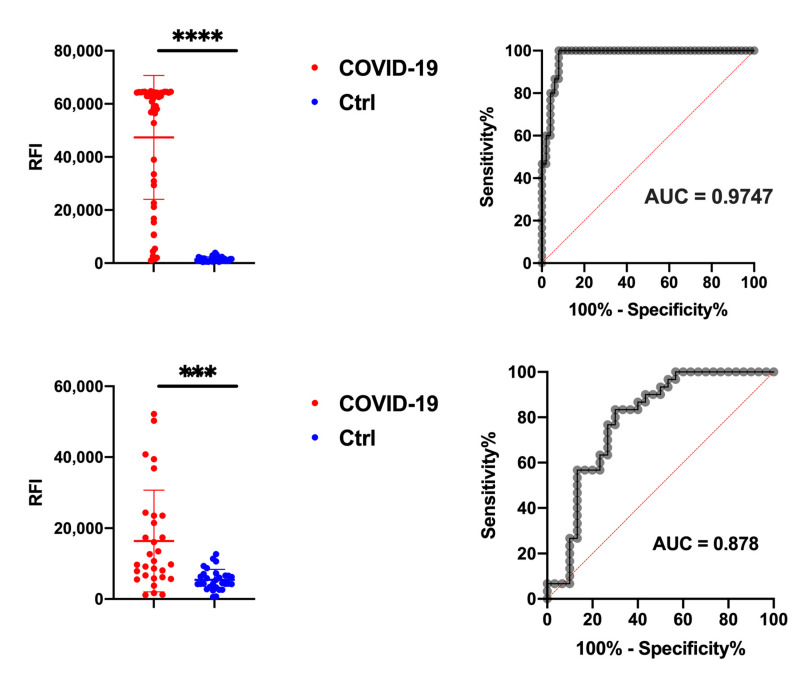
Upper panel: unpaired *t*-test results for the peptide specific IgG to AM57 probed with sera (*N* = 50) from COVID-19 patients and control patients (*N* = 50) and corresponding AUC. Lower panel: unpaired t-Test results for the peptide specific IgM to AM57 probed with sera (*N* = 30) from COVID-19 patients and control patients (*N* = 30) and corresponding AUC. Significative: *p* < 0.05; * *p* < 0.05; ** *p* < 0.01; *** *p* < 0.001; **** *p* < 0.0001.

The results of the study on *N* = 50 COVID-19 serum samples vs. *N* = 30 healthy controls of the IgG and IgM reactivity to the immunodominant AM57 epitope on N protein are summarized in Figure 4. Overall, for IgG (Figure 4, upper panel), AM57 showed an excellent diagnostic performance (*p* value < 0.0001) in discriminating the two tested groups, with 92% sensitivity and 100% specificity, almost matching the performances given by the full N antigen (see Figure S2). After ROC curve analysis, AUC is 0.9747. The lack of false negative results detected among the N = 50 Covid-19 samples suggests the absence of pre-existing cross-reactive response, likely due to low conservation of AM57 sequence among the common cold coronaviruses.

Also SARS-CoV-2 IgM immunoreactivity, which was tested in a set of N = 30 serum samples collected within the first month after symptom onset, showed a good discrimination ability (*p* value < 0.001) and AUC = 0.878. (Figure 4, lower panel). 

**Figure 5:**
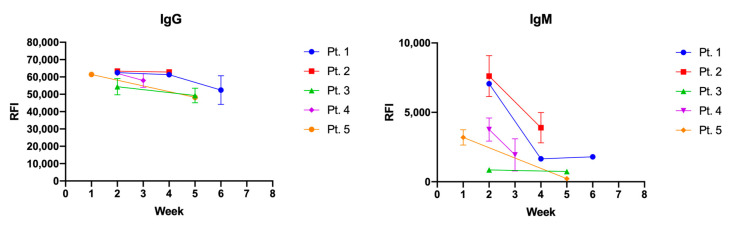
IgG (**left panel**) and IgM (**right panel**) longitudinal immune-reactivity to AM57 was evaluated on the silicon based microarray platform for site-selective peptide immobilization in a set of 5 patients sera over a period of 6 weeks after diagnosis of SARS-CoV-2 by molecular testing of nasopharyngeal swabs. Error bar is not plotted for SD values < 1000.

To evaluate the longitudinal IgG and IgM profile on AM57, immunoreactivity at different timepoints following positivity to SARS-CoV-2 PCR test was evaluated (Figure 5) in 5 patients. While the IgG response showed to be quite stable over a period of 7 weeks, as expected the IgM response showed a decline. Both IgG and IgM immune-responses were detectable at two weeks since the molecular diagnosis. Viable strategies to enhance test sensibility to favor early stage IgM/IgG response will possibly entail peptide multivalent presentation and/or switch to single-molecule detection assays. Additional optimization and further validation will be performed in our laboratories in this direction, progressively expanding the samples cohorts under examination.

Overall, the ability of the AM57 peptide to early detect IgM response worth further investigation, in line with the increasing interest on the use of peptide probes for point of care tests. The use of peptide probes in place of full antigens is particularly appealing not only for storage stability, batch-to batch consistencies and avoidance of protein refolding issues, but also for the economic sustainability of population wide testing during a pandemics. Given the quickly spread around the globe, it is anticipated that rapid serological assays for Covid-19 will be needed by an increasing number of people and over a long period of time. In this frame, synthetic peptides offer clear advantages in terms of costs and synthetic versatility allowing straightforward implementation in diverse diagnostic settings: from lateral-flow-tests for point of need diagnostics to ELISA and bead-based assays for centralized settings.

## 4. Conclusions

We here reported on a workflow for SARS-CoV-2 epitope discovery based on peptide microarrays. The discovery process started with a proteome-wide screening on high-density microarray followed by a refinement and validation phase based on low-density arrays characterized by a precise control of orientation and density of peptide probes. The results of the validation screenings showed the crucial role of testing a large number of samples and independent cohorts during a diagnostic probe refinement processes. This is particularly evident when looking at the high variability in immunoreactivity that is observable among the different sera in each set of sample. Our approach led to the identification of immunodominant regions on SARS-CoV-2 spike protein, Nucleocapsid protein and Orf1ab polyprotein. A summary study testing 50 Covid-19 serum samples highlighted the optimal diagnostic performance of a N protein linear epitope (region 155–171), which clearly outperformed other linear epitopes. High sensitivity and specificity in the discrimination of Covid-19 patients from healthy individuals were found, along with negligible cross-reactivity with common cold viruses. Early stage IgM/IgG detection remains to be improved to closely match the full N antigen performances, and it will be matter of further investigation. Overall, N protein epitope 155-171 can be considered a promising candidate for rapid implementation in cost-effective serological tests, thus expanding the tool box needed to complement Covid-19 patient assessment, vaccine research and epidemiological studies.

## Figures and Tables

**Table 1:** Demographic and clinical data on the Covid-19 patients tested.

Patients Data	Group A (Whole-Proteome Screening)	Group B (1st Microarray Screening)	Group C (2nd Microarray Screening)	Group D (Test Validation)	Group E (Longitudinal Samples)
No. patients	7	12	28	50	5
Mean age, IQR	86 (79–95)	78 (62–91)	80 (72–86)	81 (71–87)	67 (64–72)
Females	71%	58%	75%	66%	0%
Mild/moderate COVID-19	100%	92%	96%	86%	0%
Severe COVID-19	0%	8%	4%	14%	100%
Days since symptom onset, IQR	28 (27–30)	30 (25–35)	38 (28–119)	98 (28–120)	Time series (range 30–150 days)

**Table 2:** Most reactive aminoacidic sequences selected by the high-density microarray and results of the validation performed with progressively larger patients cohorts. The peptide sequences were analyzed in terms of fluorescence intensity of IgG immune-reactivity of Covid-19 positive sera and discrimination with the healthy control group by the unpaired *t* test. The *p* values of the screenings are reported.

Protein	Code	Sequence	*p* Value 1st Screening *N* = 12	*p* Value 2nd Screening *N* = 28
Orf1ab	AM48	CASYQTQTNSPRRAR	0.2958	
S	AM49	YQAGSTPCNGVEGFN	0.1942	
S	AM50	FDNPVLPFNDGVYFA	0.0180	0.0101
Orf1ab	AM51	LGVYDYLVSTQEFRY	0.1499	
Orf1ab	AM52	QDGNAAISDYDYYRY	0.0470	0.3314
Orf1ab	AM54	GLPGTILRTTNGDFL	0.2074	
S	AM55	CASYQTQTNSPRRAR	0.0359	0.2882
Orf1ab	AM56	IDLVPNQPYPNASFD	0.5966	
N	AM57	AIVLQLPQGTTLPKG	<0.0001	<0.0001
S	AM63	SNLKPFERDISTEIY	0.0249	0.2640
Orf1ab	AM65	DNQDLNGNWYDFGDF	0.0115	0.0317
N	AM66	GNFGDQELIRQGTDY	0.0771	

## Supplementary Materials

Figures S1 and S2 are available online at https://www.mdpi.com/2076-393X/9/1/35/s1.
