# Supplementary material for: SARS-CoV-2 Epitope Mapping on Microarrays Highlights Strong Immune-Response to N Protein Region
Source: Vaccines (Basel). 2021 Jan 11;9(1):35. doi: 10.3390/vaccines9010035 (PMC7827214; doi:10.3390/vaccines9010035)
Supplement: Supplementary file 1 [file vaccines-09-00035-s001.pdf]

SARS-CoV-2 epitope mapping on microarrays highlights strong immune-response to N protein region

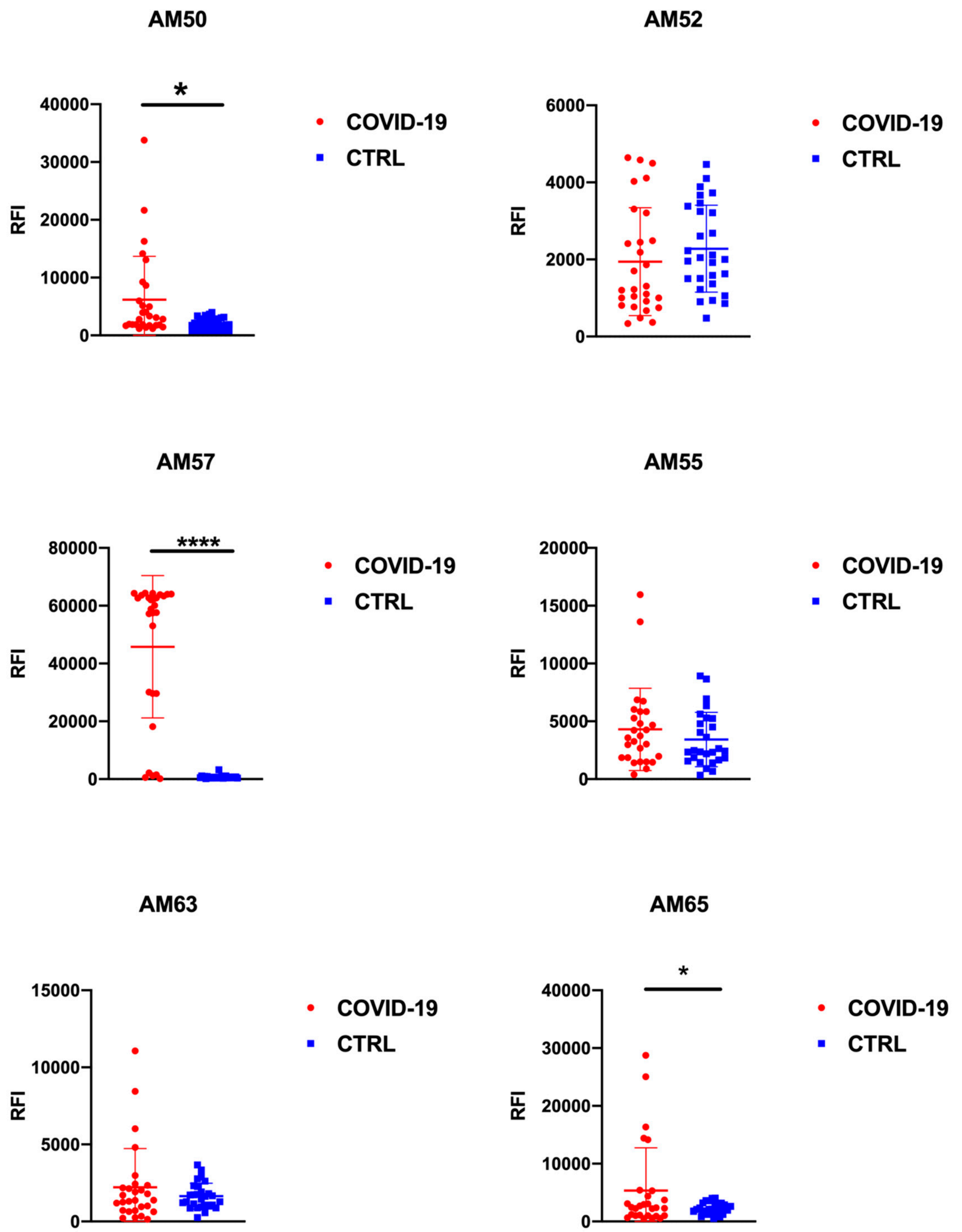

Figure S1. unpaired t-Test results for the peptide specific IgG. Peptide arrays displaying six peptide probes were probed with sera (N = 28) from COVID-19 patients and control patients (N = 28). Significative: $p < 0.05$; * = $p < 0.05$; *** = $p < 0.001$; **** = $p < 0.0001$.

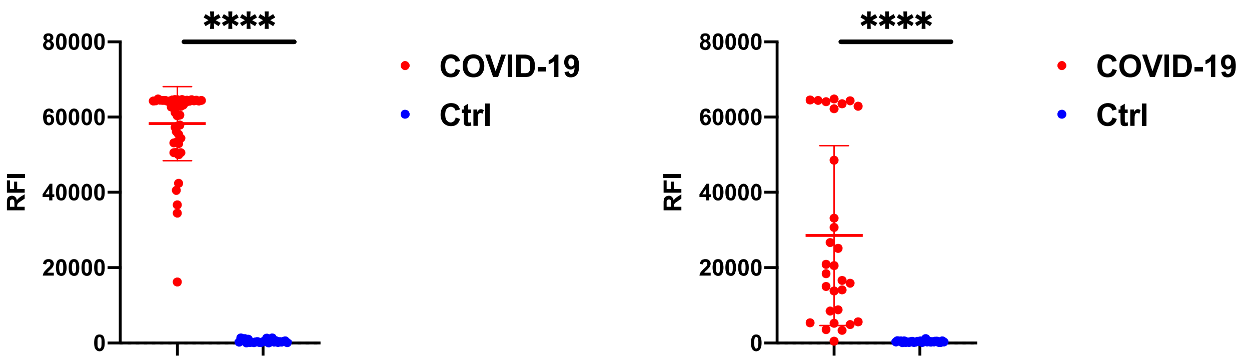

Figure S2. Left panel: IgG immunoreactivity on full N antigen. Right panel: IgM immunoreactivity on the full N antigen. **** = $p < 0.0001$.
